# Assessment of quality of edible vegetable oils accessed in Gondar City, Northwest Ethiopia

**DOI:** 10.1186/s13104-019-4831-x

**Published:** 2019-12-04

**Authors:** Yonnas Adugna Negash, Dagnachew Eyachew Amare, Bikes Destaw Bitew, Henok Dagne

**Affiliations:** Department of Environmental and Occupational Health & Safety, Institute of Public Health, College of Medicine and Health Sciences, University of Gondar, Gondar, Ethiopia

**Keywords:** Edible vegetable oil, Imported, Locally made, Quality parameters

## Abstract

**Objective:**

Edible vegetable oils are prone to quality deterioration through oxidation and microbial degradation resulting in nutritional loss and off-flavors. Quality deterioration may contribute in the formation of oxidation products that are reactive and toxic, which ultimately pose health risks including cancer and inflammation. The objective of this study was to assess quality of both imported and locally made edible vegetable oils accessed in Gondar City, Ethiopia. Cross-sectional study design was used to collect 60 samples randomly; 30 from locally made (Niger seed at market 14, Niger seed at production center 11, sunflower at the market 5) and 30 from imported palm oil brands (Avena 11, Hayat 4, Jersey 5 and Chef 10).

**Results:**

The mean value for: moisture content (%) (0.333 ± 0.08 while 0.089 ± 0.11), specific-gravity (0.823 ± 0.14 and 0.807 ± 0.115), peroxide value (15.09 ± 1.61 and 7.05 ± 0.102 mill-equivalents of oxygen/kg), acid value (2.43 ± 0.9 and 0.98 ± 0.23 mg KOH/g oil) and iodine value (115.63 ± 6.77 and 21.8 ± 3.4 g I_2_/100 g oil) for local and imported edible oils, respectively. The results highlight that all rancidity quality parameters of the locally made oil samples were not within the joint WHO/FAO standards whilst the imported oils showed a greater fatty acid saturation.

## Introduction

Edible vegetable oils are triglycerides of plant origin that include olive, palm, soybean, canola, and sunflower oil [1, 2]. Oil and fat are important nutritional components with variety of functions in our body as an energy source, membrane structures, regulating body temperature and insulate organs [3, 4].

Vegetable oils may rancid and hence lose its nutritional values and flavor upon improper extraction process, handling and storage [5, 6]. Moisture, microbes, air, anti-oxidants and exposure to sunlight are among factors determining the oils rancidity or deterioration time [7–9].

In quality control, several parameters such as iodine value (degree of unsaturation), peroxide value (formation of primary oxidation products), moisture content, specific gravity (purity), and acid value (free fatty acids formation because of rancidity) are key parameters of interest as they determine the shelf-life quality and hence the economic value of oils [3, 10]. Rancidity of vegetable oils may pose health risks including cancer and inflammation because of the formation of toxic and reactive oxidation products [2, 11, 12]. For healthy consumption, unsaturated oils are better than the saturated. Consumption of palmitic oil (highly saturated) is associated with an increased risk of developing cardiovascular diseases [12, 13]. In contrast, edible vegetable oils such as sunflower, olive, canola and Niger-seed oils contains high levels of polyunsaturated fats [2, 14] which make them susceptible for rancidity.

Unlike in developing countries like Ethiopia, the developed countries have a strict food safety regulation [5]. Studies showed that developed countries society have greater awareness compared to developing countries in edible oil purchasing choice [15–17]. WHO/FAO has outlined quality standards for various edible vegetable oils constituents; heavy metals, fatty acids composition, antioxidants, micronutrients and other physicochemical parameters [18]. The WHO/FAO guideline sets the maximum allowable limit for edible oils quality parameters including moisture (0.2%), acid value (0.6 mg potassium hydroxide/g oil) and peroxide value (10 mill-equivalents oxygen/kg oil) [19].

Due to limited published researches and its public health importance, periodic oil quality analysis is required. Therefore, the aim of the study was to assess qualities of edible vegetable oils accessed in Gondar city, Ethiopia regarding rancidity and level of fatty-acid saturation.

## Main texts

### Methods

#### Study design and sample size

A cross-sectional study was conducted on selected edible vegetable oils in the local market of Gondar city in 2019. Sixty samples; 30 from locally made (Niger seed at the market 14, Niger seed at the production center 11, sunflower at the market 5) and 30 from imported palm oil brands (Avena 11, Hayat 4, Jersey 5 and Chef 10) were taken.

#### Experimental procedure

Purchased oil brands in the local market were transported to the Department of Biology laboratory, University of Gondar for analysis. Care was taken to avoid air contact during analysis to keep-away oxidation reactions.

All the parameters analysis were performed using the standard methods of oil analysis by Paquot [20].

#### Moisture content

Ten g of oil sample was placed in a weighed crucible. The samples were dried for 1 h to constant weights in an oven set at 105 °C and then allowed to cool in desiccators for 15 min and finally, the difference was calculated using the following equation.

$$\% Moisture = \frac{W1 \times 100}{W2}$$

where, W1 = weight loss (g) upon drying, W2 = weight (g) of the oil sample.

#### Specific gravity

Dry pycnometer was used to determine specific gravity. Specific gravity were measured by Relative Density of oil to water. Distilled water was added into the pycnometer followed by measurement using electronic balance. Similarly, oil weight was measured. Care was taken to avoid leakage of air into the pycometer. The specific gravity value was calculated as follows:

$$Specific \ gravity = \frac{{{\text{Weight of the oil }}\left( {\text{g}} \right)}}{{{\text{Weight of distilled water }}\left( {\text{g}} \right) }}.$$

#### Peroxide value

Ten mL of oil sample was dissolved in acetic-acid/chloroform (3: 2 ratios) solvents. This solution was further reacted with 0.5 mL of 15% potassium iodide (KI). The liberated iodine was titrated with 0.1 N sodium-thiosulphate using 0.5 mL starch as indicator. Blank titration was performed. The peroxide value was calculated as follows:

$$Peroxide\ value = \left( {{\text{B}} - {\text{S}}} \right) \times {\text{W }} \times {\text{N}}$$

where, S = volume of sodium-thiosulphate consumed by the oil sample, B = volume of sodium-thiosulphate used for blank, W = weight of oil sample, N = the normality of sodium-thiosulphate.

#### Acid value

Mixture of 10 mL of oil sample and 100 mL of ethyl-alcohol was heated until the content started boiling. The hot content was cooled and titrated with 15% KOH solution using phenolphthalein as endpoint indicator. Acid value was calculated as follows:

$$Acid\ value = \frac{V \times N \times M.wt}{W}$$

where, V = volume of standard KOH solution in mL, N = normality of standard KOH solution, W = weight of oil sample in grams, M.wt (molecular weight) of KOH = 56.1 g/mol.

#### Iodine value

Mixture of 0.5 mL of oil sample and 10 mL chloroform was added into 25 ml of iodine solution, stayed for 30 min for a complete reaction between iodine and the unsaturated bonds of oils. The flask was covered by aluminum foil to avoid light exposure. Then, 20 mL of 15% aqueous KI and 100 mL of water was added to transform leftover iodine to iodide. The final content was titrated with 0.1 N sodium-thiosulphate (Na_2_S_2_O_3_) solutions using starch as an indicator. Iodine value was calculated as follows:

$$Iodine \ value = \frac{{\left( {{\text{A}} - {\text{B}}} \right) \times {\text{N }} \times 0.127 \times 100}}{W}$$

where, A = mL of 0.1 N Na_2_S_2_O_3_ required by oil sample, B = mL of 0.1 N Na_2_S_2_O_3_ required by the blank, N = normality of Na_2_S_2_O_3,_ W = weight of oil in gram, 1 mL 1 N Na_2_S_2_O_3_ = 0.127 g I_2._

#### Quality assurance

For quality assurance, instrument calibration and pretest for functionality of instruments were conducted before the laboratory analysis. Analysis involved blank measurements and all the measurements were done triplicate wise. Standard analysis methods were followed.

#### Data processing and analysis

Data were entered in Epi-Info-version-7.2.10 and exported to SPSS-version-20 for statistical analysis. Mean values and standard deviations were calculated. ANOVA was used for the analysis of variance among the oil brands for the respective parameters, and independent t-test was used for comparison between locally made and imported oils.

### Results

#### Quality analysis results

From the 60 samples analyzed, moisture content 9 (30%) and 25 (83.3%), specific gravity 8 (26.7%) and 7 (23.3%), peroxide value 5 (16.7%) and 20 (66.7%), acid value 4 (13.3%) and 2 (6.7%) and iodine value 8 (26.7%) and 2 (6.7%) were within the limits of WHO/FAO standards for locally made and imported edible vegetable oil, respectively (Table 1).

**Table 1:** Mean value ± standard deviation of physical–chemical characteristics of locally produced and imported edible vegetable oils collected from different sites in Gondar city, February to March, 2019

Types of product	Parameters (mean value ± standard deviation (SD))				
	Moisture content (%)	Specific gravity (unit less)	Peroxide value (mill equivalents oxygen/kg oil)	Acid value (mg KOH/g oil)	Iodine (I_2_) value (gram I_2_/100 g oil)
Niger seed at the market	0.32 ± 0.10	0.88 ± 0.07	13.84 ± 1.24	3.1 ± 1.1	129.5 ± 9.7
Niger seed at source	0.36 ± 0.11	0.79 ± 0.20	16.25 ± 2.5	3.0 ± 1.09	96.4 ± 6.8
Sunflower	0.32 ± 0.11	0.8 ± 0.15	15.2 ± 1.09	1.2 ± 0.51	121 ± 3.8
Avena	0.11 ± 0.09	0.79 ± 0.14	4.91 ± 1.18	1.0 ± 0.17	17.2 ± 2.6
Hayat	0.18 ± 0.12	0.81 ± 0.20	8.5 ± 0.98	1 ± 0.3	31.0 ± 7
Jersey	0.004 ± 0.01	0.81 ± 0.06	11.4 ± 1.06	0.9 ± 0.12	23.5 ± 2.7
Chief	0.08 ± 0.10	0.82 ± 0.06	3.4 ± 0.87	1.0 ± 0.3	15.5 ± 1.3
WHO standards	0.2%	^a^	10	^b^	^c^

^a^Specific gravity: for Niger seed 0.917–0.92, Sunflower 0.919–0.923, and palm oil 0.891–0.899 recommended by WHO/FAO

^b^Acid value: refined oil 0.6 mg potassium hydroxide (KOH)/g oil

^c^Iodine value: Niger seed 112–129, sunflower 118–141 and palm oil 50–55 (gram I_2_/100 g oil) recommended by WHO/FAO

The mean moisture value for locally made and imported oils were found to be 0.333 ± 0.08 and 0.089 ± 0.11, respectively. There is a significant difference in moisture content between local made and imported edible vegetable oils having p-value 0.016 of 95% confidence interval (CI). However, there is no significant difference within oil brands.

The mean specific gravity value for the locally made and imported oils were 0.823 ± 0.14 and 0.807 ± 0.115, respectively. There is a significant difference on specific gravity between locally made and imported edible oils with p-value < 0.001 of 95% CI. Whereas, there was no significant difference among the various brands.

The mean peroxide value for the local products and imported were 15.09 ± 1.61 and 7.05 ± 0.102, respectively. There is a significant difference being local and imported edible oil with p-value < 0.003 of 95% CI.

The mean acid value for the locally made and imported oils were 2.43 ± 0.9 and 0.98 ± 0.23, respectively. The acid value between the locally made and imported edible oils significantly differ with p-value < 0.001 of 95% of the CI. The locally made edible oils have shown a greater deviation from the WHO/FAO standard value (0.6 mg KOH/g oil). Niger seed edible oil has shown a significant difference with avena, Chef, and jersey brands with p-value < 0.001, 0.002 and 0.001, respectively of 95% CI.

The iodine value was significantly differ between locally made and imported edible oils with p-value < 0.001 of 95% CI. The iodine value for the local products and imported were 115.63 ± 6.77 and 21.8 ± 3.4, respectively. It was observed that the iodine value significantly different among the brands with a p-value < 0.001 of 95% CI.

### Discussions

Iodine value of oils was determined mainly to determine which oil types are more saturated. The results depicted that the locally made oils (sunflower and Niger seed) have displayed a higher iodine value, proportional to the unsaturated fatty acids when compared to the imported. Unsaturated fatty-acids is recommended for a healthy consumption over highly saturated fatty-acids containing oil.

Previous studies depicted that sunflower and Niger seed oils have a higher unsaturated fatty-acids compared to palm oils [21]. Hence, the observed higher iodine value in the locally made oils indicated that they are likely to be healthier for consumption. Studies have recommended to switch from saturated to unsaturated fats because of the risk of cardiovascular disease associated with high consumption of saturated fatty-acids [22–25]. However, highly unsaturated oils undertake oxidation degradation because of their double bonds [7] unless sufficient antioxidant is added [26–28].

The mean peroxide value in this study was 2.73 and 15.03 mill-equivalents of active oxygen/kg oil for imported oils and locally made oils, respectively. Peroxide values of the locally made edible oils were deviated significantly (P < 0.05) from the WHO/FAO limits (10 mill-equivalents of active oxygen/kg oil). The lower peroxide value for the imported oils is likely attributed to their higher saturation, which resists oxidation. Moreover, palm oils are known for having antioxidants (vitamin E) that extend its shelf-life [10, 29].

When moisture content ranges from 0.05 to 0.3 in edible oils, it shows that rancidity likely to occur [29]. The maximum allowed moisture content in edible oils is 0.2% [14]. The higher moisture content observed in the local products could be the poor moisture refining process as the companies are using low technology for oil production [30]. The other reason could be that those locally made oils require extra heating for reducing the moisture content kept inside the seed [30, 31] before production. Previous studies have found that oils which were produced using low technology displayed a higher moisture [31, 32]. Therefore, the higher moisture content in the locally made oils indicated that they are likely to undergo rancidity. This is because the presence of sufficient amount of moisture favors microbial growth [31]. Previous studies have shown that fungus species such as *Aspergillus niger* and *Mucor* species survive and reproduce when the moisture content value is higher than 0.2% [29].

The acid values obtained in this study were 2.728 mg KOH/g for the locally made oils and 0.999 mg KOH/g for the imported oils which showed that both were above the permissible level (0.6 mg KOH/g) [18]. The local edible oils have displayed a higher acid value than the imported products where the formers higher fatty unsaturation could be responsible for the deviation. Consuming rancid edible oil is unlikely to cause immediate health impact, but can reduce significantly the nutritional value of foods by degrading the essential fatty-acids and nutrients [22, 33]. The acid value of palm oil brands in this study is in line with the previous study [34].

In this study, the mean value (0.823) of specific gravity for locally made oils was not in line with the WHO limit (Niger seed 0.917–0.92; Sunflower 0.919–0.923) [18]. The specific gravity value which was significantly deviated from the standards could be related to the poor refining and upgrading process in the local production facilities. Insufficient refining process may lead for a higher impure oil grade [14, 29]. Furthermore, the specific gravity values of the locally made oils were below the standard ranges make them susceptible to adulteration [30].

### Conclusions

The results showed that locally made edible oils displayed higher degree of rancidity compared to the imported. The higher iodine value in the locally made oils indicated that these oil types are better for public consumption with respect to health risks.

## Limitation of the study

Comparison of oil qualities between oils taken from the production facility and oils taken in the market were only performed for the locally made oil types.

## Data Availability

The dataset is accessible at the corresponding author upon request.

## Abbreviations

ANOVA: analysis of variance
CI: confidence interval
FAO: Food and Agriculture Organization
I_2_: iodine
KI: potassium iodine
KOH: potassium hydroxide
SD: standard deviation
Na_2_S_2_O_3_: sodium thiosulfate
WHO: World Health Organization
